# Accuracy of dynamic computer-assisted implant surgery across different degrees of edentulism: does experience matter? An in vitro study

**DOI:** 10.1186/s40729-025-00648-0

**Published:** 2025-09-22

**Authors:** Mats Wernfried Heinrich Böse, Alexander Jung, Florian Beuer, Stefano Pieralli, Moritz Nensa, Maria Bruhnke

**Affiliations:** 1 Department of Prosthodontics, Geriatric Dentistry and Craniomandibular Disorders, Charité – Universitätsmedizin Berlin, corporate member of Freie Universität Berlin, Humboldt-Universität zu Berlin, Berlin Institute of Health, Aßmannshauser Str. 4-6, 14197 Berlin, Germany; 2 Private Dental Office 71 | ZWEI, Außer der Schleifmühle 71, 28203 Bremen, Germany; 3 Private Dental Office Dr. Carolin Röder, Friedrich-Ebert-Str. 103, 46535 Dinslaken, Germany

**Keywords:** Dental implants, Computer-assisted surgery, Computer-aided surgery, CAD/CAM, Virtual patient, Backward planning

## Abstract

**Purpose:**

To evaluate the accuracy of a dynamic computer-assisted implant surgery (dCAIS) system utilized by two clinicians with differing levels of surgical experience in implant dentistry.

**Methods:**

A total of 72 conical dental implants were placed into 18 jaw models using a dCAIS system (Navident, ClaroNav, Toronto, Canada). Implant planning and placement were performed by both a novice and an experienced implantologist, following a standardized protocol across single-tooth gaps, shortened dental arches, and fully edentulous jaws. Pre- and postoperative CBCT scans were aligned, and deviations were analyzed at the coronal entry point (2D), apex (3D), apex vertically (V) and in angular deviation (°). Level of significance was set at *p* < 0.05.

**Results:**

Mean deviations across all implants placed were: 1.25 ± 0.90 mm at entry point (2D), 1.66 ± 0.68 mm at apex (3D), 0.70 ± 0.51 mm at apex (V) and 4.96 ± 3.16° angular. No statistically significant differences were found between clinicians, jaws, or sides (*p* > 0.05). However, a significant correlation was observed regarding accuracy at entry points and angular deviations, indicating lower accuracy in fully edentulous jaws (*p* < 0.05).

**Conclusions:**

The investigated dCAIS system enabled accurate transfer of the planned to the actual (in vitro) implant position independent of operator experience. dCAIS systems may help mitigate the impact of limited surgical experience and contribute to more predictable outcomes. However, reduced anatomical reference points in edentulous jaws justify further clinical investigation and workflow optimization.

## Background

Dental implantology is undergoing consistent transformation by advances in digital technologies and the demand for prosthetically driven implant placements reducing the risk of technical, biological and esthetic complications [1–6]. The increasing expectations of practitioners and patients regarding long-term function, esthetics, and comfort of implant-supported restorations required the development of more predictable surgical protocols over the years [3, 7–9]. A key aspect to these developments is computer-assisted implant surgery (CAIS), which enables the creation of a virtual three-dimensional (3D) patient in dedicated implant planning software [10]. CAIS enables ideal virtual implant planning and, successively, accurate transfer to the patient´s mouth [5, 11–13]. Thereby, three approaches have emerged: static computer-assisted implant surgery (sCAIS) [14], dynamic computer-assisted implant surgery (dCAIS) [15] and robotic computer-assisted implant surgery (rCAIS) [13]. They differ in terms of the techniques used to guide implant placement, the degree of intraoperative flexibility, and the hardware required [13–15]. Among dynamic navigation systems, the X-Guide (X-Nav Technologies, Lansdale, PA, USA), Navident (ClaroNav, Toronto, Canada), and the Straumann Falcon (Straumann Group, Basel, Switzerland) represent commonly used approaches. While X-Guide uses stereoscopic cameras with an X-Point tracking clip and Navident employs tracer-based optical registration, the Falcon integrates motorized optical tracking with touch-screen control and optional smart glasses. All systems share the aim of enhancing accuracy and intraoperative flexibility but differ in registration methods, hardware design, and digital workflow integration. However, all approaches share the common goal of improving implant dentistry, reducing operator-dependent variability, and supporting prosthetically driven treatment concepts.

Dynamic computer-assisted systems utilize real-time navigation based on optical tracking for accurate implant positioning [5, 12, 16, 17]. These systems allow intraoperative adjustments and visual feedback, enabling clinicians to react to unforeseen anatomical challenges since no surgical guides are necessary [18, 19]. Additionally, dCAIS ensures continuous cooling of the drills while requiring less intraoral space than sCAIS [18, 19] and offering comparable results regarding the accuracy of sCAIS and rCAIS [5, 13–15]. However, a learning curve has been documented in current literature and is part of ongoing scientific studies [12, 16, 20–22].

Whereas dental implantology was traditionally reserved for more experienced practitioners, it is now increasingly being integrated into under- and postgraduate training [23–26]. This development is likely linked to the continuous rise in the number of implants placed [27, 28]. Consequently, it has become necessary for implantology training to be incorporated into under- and postgraduate curricula, so that its advantages and limitations are understood early on and practitioners become familiar with its application and risks. Although current literature indicates that the use of sCAIS or dCAIS may enhance accuracy among less experienced implantologists, the available data remain limited [24, 26]. Accordingly, further studies should investigate the influence of individual experience on the accuracy of dCAIS.

Thus, the aim of this in vitro study was to evaluate the accuracy of dCAIS comparing a novice and experienced implantologist. Furthermore, it should be evaluated whether the degree of edentulism (single-tooth gap, shortened dental arch, or complete edentulism) may have a potential impact on accuracy. The null hypothesis was that there is no statistically significant difference depending on practitioners and degree of edentulism.

## Methods

### Study design

The present study was designed following the Checklist for Reporting in vitro Studies (CRIS guidelines) [29] and CONSORT (Consolidated Standards of Reporting Trials) statement [30] where applicable. According to the local ethics committee, no ethical approval was necessary due to the in vitro design with artificially produced jaws. Sample size calculation was conducted regarding the provision and placement of 72 dental implants in 18 artificial jaws (four in each jaw) using G*Power (version 3.1.9.7, Heinrich Heine University, Duesseldorf, Germany) [31, 32]. Considering an allocation of 1:1, a statistical power of 80% and an effect size of d = 0.7, a total of 34 implants should be placed per practitioner (group) to ensure a significance level of *p* < 0.05. One examiner without clinical experience regarding dental implant placements (A.J.) and another examiner with a certified focus of activity in dental implantology, over five years of experience as an implantologist, experience with 100 in vitro cases and 10 clinical cases regarding the investigated dCAIS system (M.W.H.B.) performed surgeries. Figure (Fig.) 1 visualizes a corresponding flow chart.

**Fig. 1:**
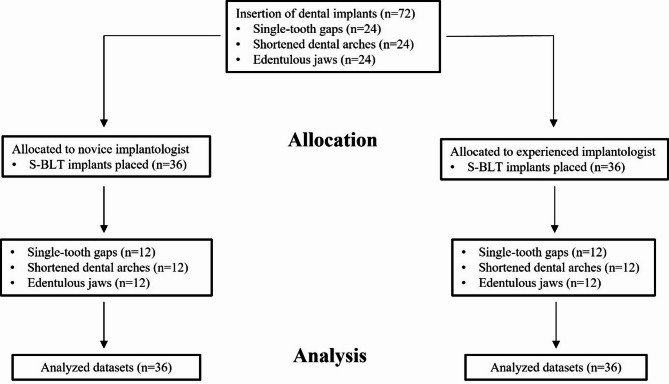
Flow chart visualizing the present study design. Abbreviation: S-BLT (Straumann Bone Level Tapered)

### Experimental set-up

In total, nine pairs of artificial jaws were fabricated by an external company using injection-molding technique (Sawbones, Sawbones Europe AB, Malmö, Sweden). According to the manufacturer, the polyurethane-based material approximates cortical and cancellous bone densities within a standardized range. However, it does not fully reproduce the heterogeneous trabecular architecture, viscoelasticity, or the wide spectrum of clinical bone qualities described by Lekholm and Zarb. To improve the visualization in cone-beam computed tomography (CBCT), models were additionally coated with zinc powder

**Fig. 2:**
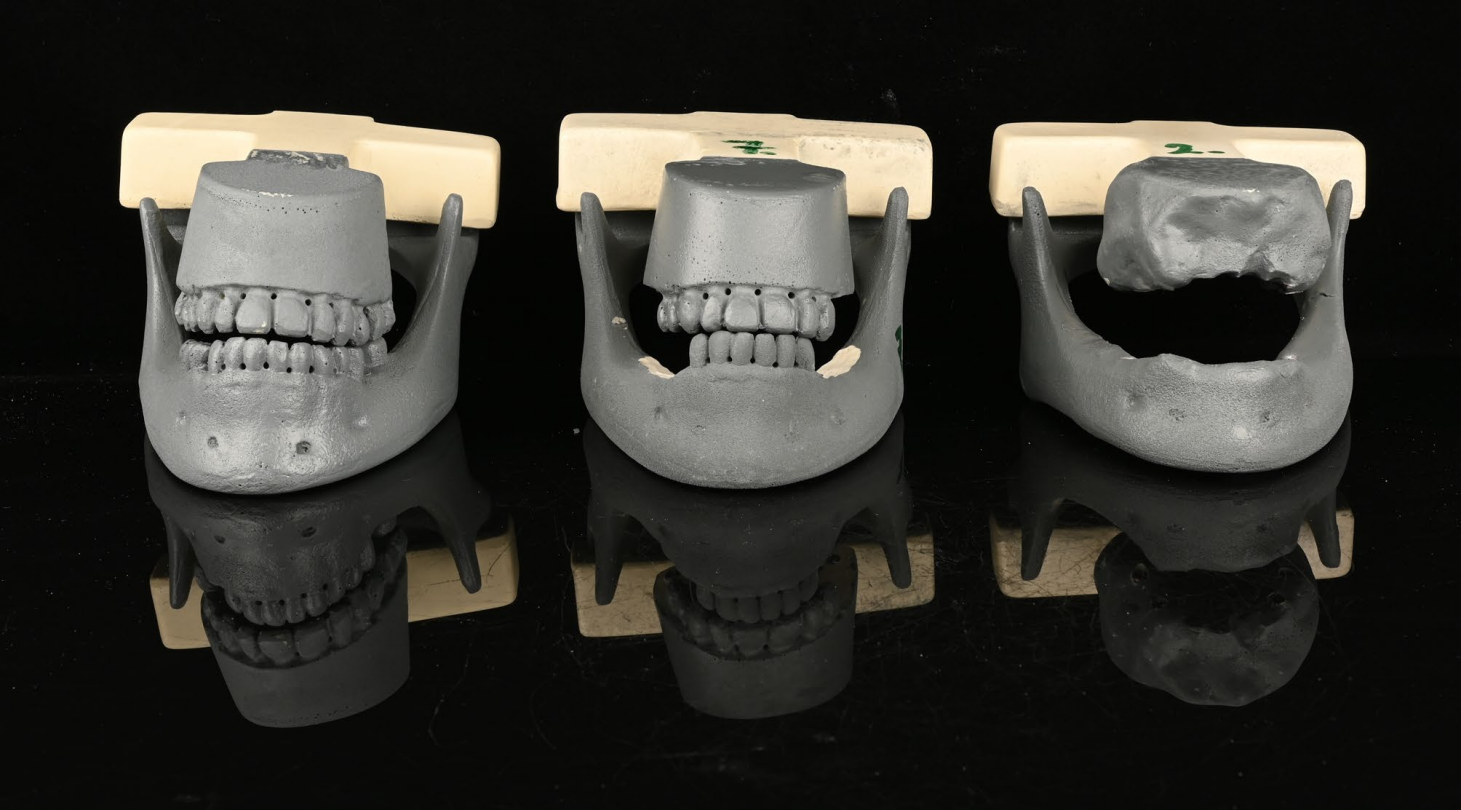
Artificial jaw pairs with single-tooth gaps (left), shortened dental arches (middle) and full edentulism (right)

Of the nine jaw pairs, three were completely edentulous, while six were fully dentate, excluding third molars, as defined by the Fédération Dentaire Internationale (FDI) numbering system: regions 17-27 and 37-47. Therefore, to enable a comparative analysis of implant deviations regarding single-tooth gaps, shortened dental arches and fully edentulous jaws, selected teeth were manually removed from the dentate models using a handpiece and metal bur (Fig. 2). The specific teeth designated for removal were determined by the author M.W.H.B. using an online randomization tool (https://www.randomizer.org, last accessed 16th of June, 2022)

**Fig. 3:**
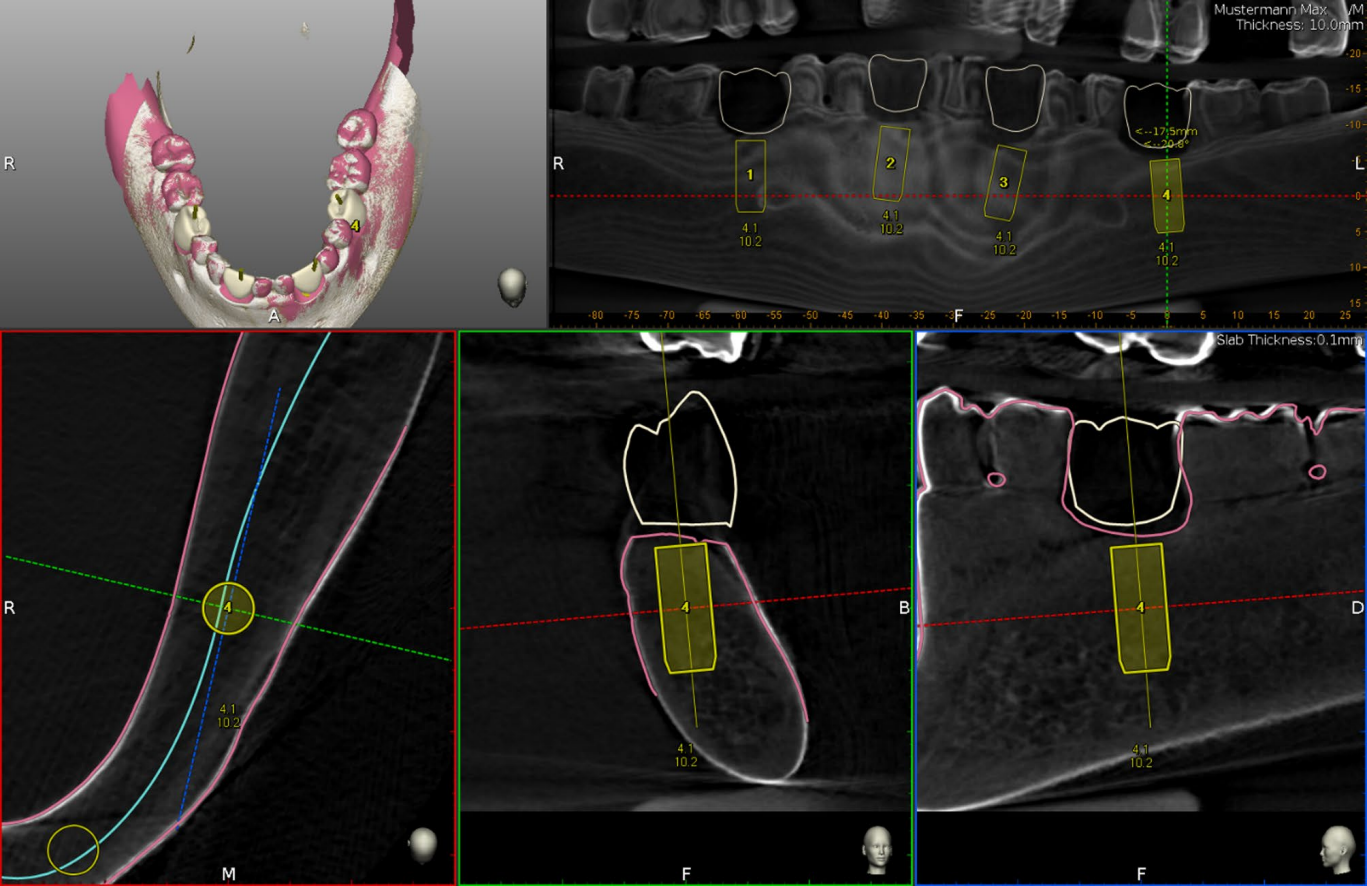
Exemplary digital implant planning in the IPS of the investigated dCAIS system. Abbreviations: IPS (implant planning software), dCAIS (dynamic computer-assisted implant surgery), CBCT (cone-beam computed tomography); grey shades: CBCT data, yellow: virtually planned implants (4.1 × 10 mm), white: digital wax-ups, pink: scan data

**Fig. 4:**
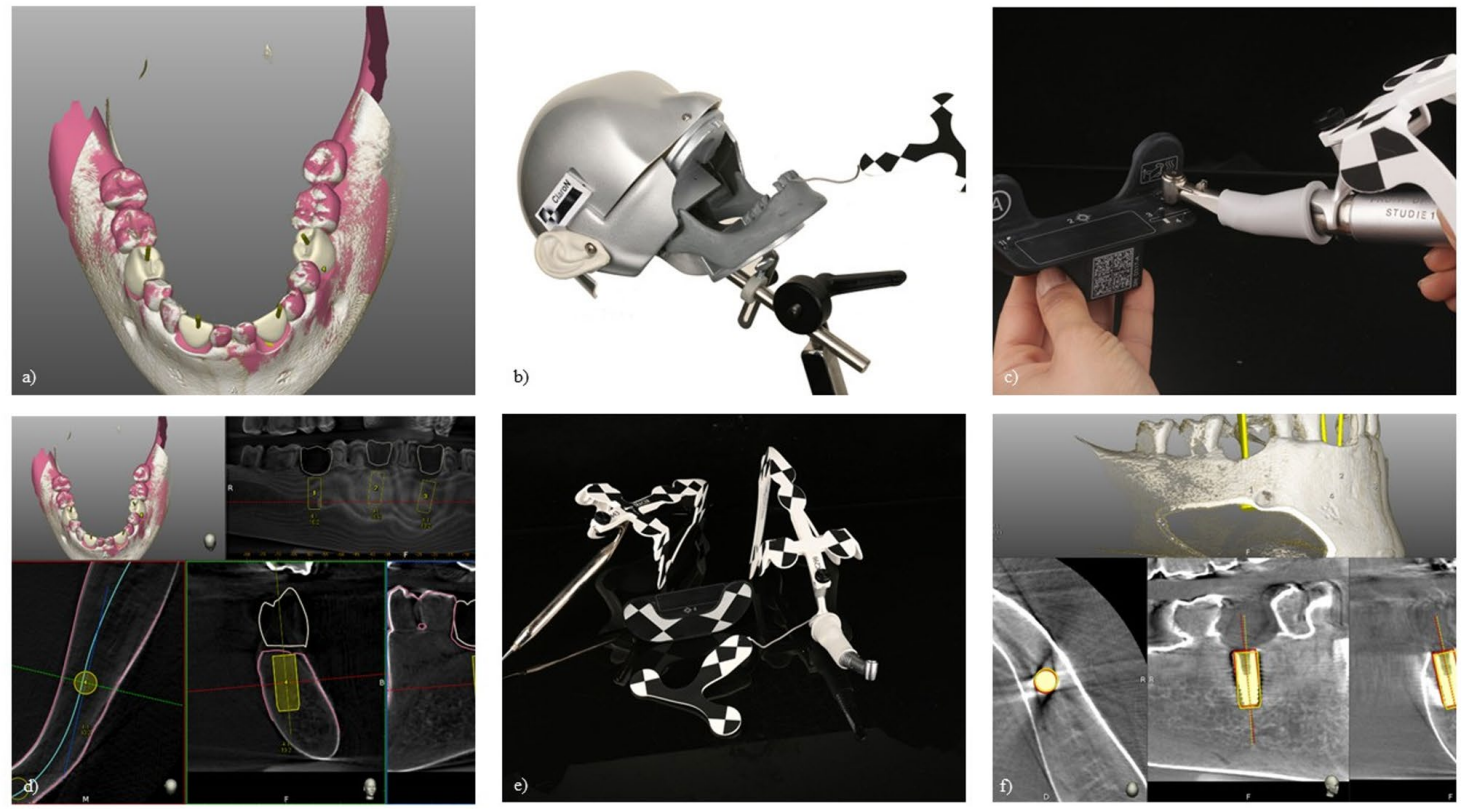
Illustrated overview of the workflow. (**a**) and **d**) digital implant planning, (**b**) artificial jaw with optical reference body fixated in phantom head, (**c**) registration tool with mounted surgical hand piece, **e**) top left: ‘tracer’ with mounted reference body, top right: surgical hand piece with mounted reference body, middle: registration tool, bottom: reference body for lower jaw, **f**) matching of pre- and postoperative CBCT datasets [41]

Afterwards, CBCTs, (Veraviewpocs 3D R100, J. Morita Europe GmbH, Dietzenbach, Germany) with a field of view, (FOV) of 80 × 80 cm, 90 kV and 7 mA were made of all jaw pairs. To ensure the same alignment while delivery of pre- and post-operative CBCTs, non-radiopaque silicone was used for fixation. Resulting DICOM, (Digital Imaging and Communications in Medicine) datasets were imported into the implant planning software, (IPS) of the dCAIS system to be investigated, (Navident 3, ClaroNav, Toronto, Canada). This resulted in nine virtual in vitro patients, (*n* = 9), each consisting of an upper and lower jaw, (*n* = 18). Digital wax-ups were generated to simulate prosthetically driven implant placements and occlusal screw-retention. Subsequently, *n* = 36 ⌀4.1 × 10 mm bone level titanium implants, (Straumann Bone Level Tapered [S-BLT], Straumann GmbH, Basel, Switzerland) were virtually planned and positioned by the two operators each, (Figs. 3 and 4a and d)

Twenty-four implants were planned and subsequently installed into single-tooth gaps, 24 into shortened dental arches and 24 into fully edentulous jaws (*n* = 72). Thereby, the sequence of implant placements in each jaw was again randomized in accordance with CRIS guidelines [25], using the same online tool (https://www.randomizer.org, last accessed 31st of January 2023).

For the implementation of the study, artificial jaws with the required optical reference body were fixed to a phantom head (Fig. 4b). The investigated dCAIS system was then positioned to enable real-time tracking via its high-performance camera system. Additional reference bodies with a specific black-and-white pattern were mounted on both the surgical handpiece and the so-called ‘tracer’ device (Fig. 4e). Thereby, all instruments required individual recognition and calibration by dCAIS cameras utilizing a designated registration tool (Fig. 4c and e). The tracing procedure, which is essential for real-time tracking with the investigated dCAIS was initiated using the ‘tracer’. Distinct anatomical landmarks (e.g., cusp tips) were identified and scanned by moving the ‘tracer’ tip over and around the marked areas, including adjacent surfaces. Thereby, dCAIS software provided real-time feedback on the number of points required to achieve sufficient registration, allowing the operator to adjust the number of registration points accordingly. Throughout tracing, continuous contact between the tracer tip and the artificial jaw surface was maintained.

Upon completion of tracing, the surgical handpiece and each drill were calibrated prior to use. Drilling speeds were set based on the surgical protocol and manufacturer recommendations:

- Marking of implant site: rose drill Ø2.3 mm at 800 rpm.
- Initial preparation: BLT pilot drill Ø2.2 mm at 800 rpm.
- Intermediate preparation: BLT drill Ø2.8 mm at 600 rpm.
- Final preparation: BLT drill Ø3.5 mm at 500 rpm.

Subsequent implant insertion was performed machine-driven and fully guided using the surgical handpiece and registration tool.

### Accuracy assessment

**Fig. 5:**
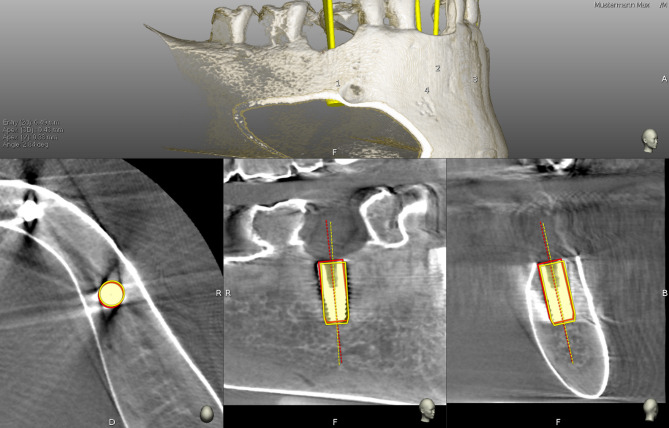
Superimposition of pre- and postoperative CBCT datasets. Abbreviations: CBCT (cone-beam computed tomography); grey shades: CBCT data; yellow: planned implant position, red: transferred implant position, top left: calculated global deviations

Following implant placement, a second CBCT scan was acquired for each jaw pair using identical parameters and utilizing silicone fixations from preoperative scans, to evaluate the transferred implant positions. Resulting DICOM datasets were imported into the corresponding digital implant planning files of IPS. The IPS of the investigated dCAIS includes a built-in tool for comparing the planned with the transferred implant position. To enable this comparison, an independent examiner was required to manually identify and confirm six anatomically corresponding landmarks on both the pre- and postoperative CBCT scans. Furthermore, the implant geometry (including diameter and length) used during planning was superimposed onto the postoperative scan and manually aligned with the radiographic representation of the implant (Figs. 4f and 5).

Subsequently, software automatically computed the following absolute deviations:

1. Coronal deviation in mm (two-dimensional, 2D).
2. Apical deviation in mm (3D).
3. Apical deviation in mm (vertical, V).
4. Angular deviation in degrees (°).

### Statistical analysis

Statistical analysis was performed by an independent examiner using SciPy (SciPy Developers), a Python-based open-source software used for scientific computing. Prior to further analysis, the data were tested for normality and zero mean using the Kolmogorov–Smirnov test. The results confirmed that all variables were normally distributed and centered around zero. Subsequently, the documented deviations were analyzed to assess potential systematic differences based on two different operators with different experience, jaw region (maxilla vs. mandible), and quadrant location (left [II. & III.] vs. right [I. & IV.]). In addition, Spearman rank correlation coefficients were calculated to explore potential associations between gained operator experience (order of placement) within this study and implant accuracy across different jaw pairs. Based on the sample size calculation, the level of statistical significance was set at *p* < 0.05.

## Results

Implant placements proceeded without complications in accordance with the protocol described and all acquired datasets were suitable for analysis. The mean deviation calculated by the integrated software at the implants entry point (2D) was 1.16 ± 1.04 mm (range: 0.08–4.42 mm) for the novice and 1.33 ± 0.73 mm (range: 0.33–2.74 mm) for the experienced operator. For the implants apex (3D) and the implants apex (V) deviations were 1.52 ± 0.67 mm (range: 0.22–2.97 mm) and 0.76 ± 0.58 mm (range: 0.01–2.79 mm) for the novice and 1.80 ± 0.67 mm (range: 0.61–3.56 mm) and 0.63 ± 0.43 mm (range: 0.00–2.13 mm) for the experienced implantologist, respectively. The median angular deviation was 5.02 ± 3.36° (range: 0.00–13.14°) for the novice and 4.90 ± 3.00° (range: 0.73–15.33°) for the experienced surgeon. No statistically significant differences could be documented between the operators (*p* > 0.05, Table 1, Tab.) at all measurement points resulting in the following mean deviations for all 72 implants placed:

**Table 1:** Comparison of operators’ experience and respective *p*-values

	Mean deviations and (SD) in mm and °		
	novice	experienced	*p*-values
Entry point (2D)	1.16 (1.04)	1.33 (0.73)	0.45
Apex (3D)	1.52 (0.67)	1.80 (0.67)	0.08
Apex (V)	0.76 (0.58)	0.63 (0.43)	0.29
Angle	5.02 (3.36)	4.90 (3.00)	0.88

Abbreviations: SD (standard deviation), 2D (two-dimensional), 3D (three-dimensional), V (vertical)

1. Mean coronal deviation (2D): 1.25 ± 0.90 mm (range: 0.08–4.42 mm).
2. Mean apical deviation (3D): 1.66 ± 0.68 mm (range: 0.22–3.56 mm).
3. Mean apical deviation (V): 0.70 ± 0.51 mm (range: 0.00–2.79 mm).
4. Mean angular deviation: 4.96 ± 3.16 mm (range: 0.00–15.33°).

All values are also documented in detail in Table 2.

Additionally, statistical analysis comparing deviations between the maxilla and mandible (Table 3), as well as between the left and right sides (i.e., quadrants II. & III. vs. I. & IV., Table 4), revealed no statistically significant differences (all *p* > 0.05). However, Spearman rank coefficients could document a significant correlation regarding the experience (order of placements) and jaw pairs. Although the implants in the fully edentulous jaws were placed at last and when both operators had gained the most experience with the experimental setup, statistically significant differences were observed in terms of deviations at coronal entry point (*p* = 0.001, *r* = 0.369^1^) regarding operators experience and angular deviation (*p* = 0.003 and *r*=-0.339) regarding jaw pairs. Although these correlations reached statistical significance, their effect sizes should be interpreted as only moderate according to Cohen’s classification. Consequently, the clinical relevance of these findings is limited, and they should not be overemphasized.

**Table 2:** Study specific data regarding the present workflow and deviations

									Deviations				
Jaw pair	Jaw		Position (FDI)		Quadrant	Operator		Order of placement		Entry point (2D, in mm)	Apex (3D, in mm)	Apex (V, in mm)	Angle (in °)
1	maxilla		15		1	novice		28		1.84	1.97	0.57	6.63
1	maxilla		13		1	novice		27		0.34	0.88	0.47	3.50
1	maxilla		23		2	novice		26		1.67	2.34	0.43	8.50
1	maxilla		25		2	novice		25		0.78	2.19	0.36	9.54
1	mandible		45		4	novice		32		0.71	2.97	2.79	7.78
1	mandible		43		4	novice		31		3.33	2.14	1.33	9.60
1	mandible		33		3	novice		30		4.42	2.37	1.10	13.14
1	mandible		35		3	novice		29		3.66	2.72	0.79	7.53
2	maxilla		15		1	experienced		25		1.82	2.10	0.46	1.73
2	maxilla		13		1	experienced		26		1.08	1.55	0.19	2.64
2	maxilla		23		2	experienced		27		1.20	1.24	0.22	2.10
2	maxilla		25		2	experienced		28		1.66	1.79	0.76	1.22
2	mandible		45		4	novice		36		1.01	0.75	0.57	6.40
2	mandible		43		4	novice		35		2.60	1.52	0.08	6.59
2	mandible		33		3	novice		34		1.73	1.12	0.89	12.74
2	mandible		35		3	novice		33		0.67	0.63	0.49	5.17
3	maxilla		15		1	experienced		29		0.87	1.89	0.18	5.76
3	maxilla		13		1	experienced		30		0.34	1.40	0.45	9.36
3	maxilla		23		2	experienced		31		1.89	1.41	0.41	4.17
3	maxilla		25		2	experienced		32		0.87	1.72	0.78	4.68
3	mandible		45		4	experienced		33		1.51	1.81	1.37	4.49
3	mandible		43		4	experienced		34		1.13	1.38	0.72	4.36
3	mandible		33		3	experienced		35		1.27	1.24	0.48	5.24
3	mandible		35		3	experienced		36		2.72	2.39	0.93	2.98
4	maxilla		11		1	experienced		1		0.33	1.30	0.64	5.21
4	maxilla		13		1	experienced		2		0.66	1.24	0.66	2.31
4	maxilla		23		2	experienced		3		0.63	1.24	0.35	3.21
4	maxilla		26		2	experienced		4		1.48	1.20	0.66	2.75
4	mandible		45		4	novice		4		0.49	0.43	0.38	2.84
4	mandible		42		4	novice		3		1.20	1.32	0.71	2.85
4	mandible		32		3	novice		2		0.79	1.64	0.09	8.73
4	mandible		35		3	novice		1		0.76	1.12	0.67	8.35
5	maxilla		14		1	experienced		5		2.74	1.59	0.76	7.85
5	maxilla		12		1	experienced		6		1.53	2.84	1.34	15.33
5	maxilla		21		2	experienced		7		0.42	2.31	2.13	4.53
5	maxilla		24		2	experienced		8		1.83	3.25	0.51	7.80
5	mandible		46		4	experienced		9		1.36	2.88	0.56	9.85
5	mandible		44		4	experienced		10		0.35	1.35	0.39	7.36
5	mandible		31		3	experienced		11		0.65	0.61	0.00	5.85
5	mandible		36		3	experienced		12		2.63	0.95	0.32	9.84
6	maxilla		13		1	novice		12		0.46	1.37	1.05	3.32
6	maxilla		12		1	novice		11		0.45	1.57	1.50	0.00
6	maxilla		24		2	novice		10		0.08	0.94	0.93	0.00
6	maxilla		26		2	novice		9		0.87	1.67	0.93	3.3
6	mandible		46		4	novice		8		0.15	1.37	0.01	7.24
6	mandible		31		3	novice		7		0.69	1.19	0.14	5.35
6	mandible		34		3	novice		6		0.45	0.59	0.04	1.32
6	mandible		36		3	novice		5		0.10	0.22	0.07	0.67
7	maxilla		17		1	novice		16		0.20	1.79	1.63	2.99
7	maxilla		16		1	novice		15		0.64	1.48	1.11	3.52
7	maxilla		24		2	novice		14		1.36	1.18	1.03	4.75
7	maxilla		27		2	novice		13		0.98	1.05	0.66	1.36
7	mandible		46		4	novice		20		1.11	1.92	1.62	4.81
7	mandible		44		4	novice		19		0.50	1.85	1.55	2.90
7	mandible		34		3	novice		18		1.37	1.20	0.60	1.94
7	mandible		35		3	novice		17		2.74	2.71	0.86	1.09
8	maxilla		17		1	experienced		13		0.66	1.51	1.00	6.39
8	maxilla		16		1	experienced		14		0.77	1.72	1.09	4.37
8	maxilla		24		2	experienced		15		1.17	2.28	1.31	4.00
8	maxilla		26		2	experienced		16		2.69	2.64	0.45	1.23
8	mandible		47		4	novice		24		1.88	2.25	0.32	3.01
8	mandible		45		4	novice		23		1.09	1.41	0.46	2.91
8	mandible		34		3	novice		22		0.16	0.82	0.29	3.49
8	mandible		36		3	novice		21		0.62	1.85	0.92	6.93
9	maxilla		16		1	experienced		17		0.61	1.26	0.41	3.31
9	maxilla		14		1	experienced		18		0.45	1.15	0.65	4.77
9	maxilla		24		2	experienced		19		1.09	1.59	0.79	6.31
9	maxilla		27		2	experienced		20		1.40	1.35	0.38	0.73
9	mandible		46		4	experienced		21		2.59	3.56	0.56	5.36
9	mandible		45		4	experienced		22		1.54	2.41	0.84	4.45
9	mandible		35		3	experienced		23		1.53	2.18	0.05	4.23
9	mandible		36		3	experienced		24		2.24	2.35	0.03	0.79
								**MEAN**		1.25	1.66	0.70	4.96
								**MEDIAN**		1.09	1.54	0.62	4.47
								**MIN**		0.08	0.22	0.00	0.00
								**MAX**		4.42	3.56	2.79	15.33
								**SD**		0.90	0.68	0.51	3.16

Abbreviations: FDI (Fédération Dentaire Internationale), 2D (two-dimensional), 3D (three-dimensional), V (vertical), MIN (minimum value), MAX (maximum value), SD (standard deviation)

**Table 3:** Comparison of maxilla vs. mandible and respective *p*-values

	Mean deviations and (SD) in mm and °		
	maxilla	mandible	*p*-values
Entry point (2D)	1.05 (0.66)	1.44 (1.06)	0.07
Apex (3D)	1.67 (0.54)	1.65 (0.81)	0.89
Apex (V)	0.76 (0.44)	0.64 (0.58)	0.33
Angle	4.42 (3.11)	5.51 (3.16)	0.15

Abbreviations: SD (standard deviation), 2D (two-dimensional), 3D (three-dimensional), V (vertical)

**Table 4:** Comparison of left (II. & III.) vs. right (I. & IV.) and respective *p*-values

	Mean deviations and (SD) in mm and °		
	right (I. & IV.)	left (II. & III.)	*p*-values
Entry point (2D)	1.10 (0.80)	1.39 (0.98)	0.17
Apex (3D)	1.71 (0.64)	1.60 (0.72)	0.50
Apex (V)	0.81 (0.57)	0.59 (0.43)	0.07
Angle	5.19 (2.93)	4.75 (3.39)	0.55

Abbreviations: SD (standard deviation), 2D (two-dimensional), 3D (three-dimensional), V (vertical); Roman numerals I. – IV. indicate respective quadrants

## Discussion

The present in vitro study evaluated the accuracy of a commercially available dCAIS system, utilized by two clinicians with differing levels of surgical experience in dental implantology. No statistically significant differences were found with respect to operator experience, jaw location, or implant position (all *p* > 0.05). However, correlation analysis revealed a significant interdependence between decreasing accuracy and in vitro scenarios specifically in terms of entry point and angular deviation (*p* < 0.05). Therefore, the null hypothesis was partially rejected.

Overall, the documented deviations fall within the range of mean values in systematic reviews [12, 15, 33]. For comparison, in 2021 Schnutenhaus et al. reported linear deviations at entry point (2D) of a mean 1.20 mm (vs. 1.25 mm in this study), global deviation at apex (3D) of a mean 1.05 mm (vs. 1.66 mm), vertical deviations at apex (V) of a mean 0.9 mm (vs. 0.70 mm), and angular deviations of a mean 4.40° (vs. 4.96°) in in vitro studies on dCAIS systems conducted between 2005 and 2020 [15]. However, it is important to note the heterogeneity of these findings, as a total of nine different dCAIS systems were evaluated across eleven included in vitro studies. Accordingly, comparative data reported by Wei et al. (2021) for the Navident system indicated mean deviations of 1.04 mm at the entry point, 1.57 mm at the apex, and 3.74° in angulation. Thus, current literature suggests that comparable accuracy values have been reported across different dCAIS systems and seem to be reliable.

It was observed that both operators were able to achieve comparable levels of accuracy (*p* > 0.05). Additionally, regarding the maximum outliers, as presented in Table 2, two were associated with the inexperienced and two with the more experienced clinician. Accordingly, the present dCAIS system appears to be capable of compensating for several years of implantological experience in terms of transfer accuracy and is in line with results presented by Wang et al. in 2022 [26]. However, it is important to consider outliers within this study, exhibiting deviations of up to 4.42 mm at the implants’ entry point and angular discrepancies reaching up to 15.33°.

Based on these findings, further statistical analyses were conducted using the entire dataset without differentiation by operator. Thereby, no statistically significant differences could be documented comparing maxilla and mandible, or the right and left quadrants. However, correlation analysis revealed a significant interdependence between decreasing accuracy and jaw pairs, specifically in terms of entry point and angular deviation (*p* < 0.05). Since all implants were initially placed in single-tooth gaps, followed by free-end situations and only subsequently in fully edentulous jaws, the degree of dentition appears to influence placement accuracy. While evidence regarding dCAIS remains considerably smaller compared to sCAIS [15, 33, 34], data concerning differences between partially dentate and edentulous jaws are scarce. The present study suggests that implant placement accuracy may decrease as intraoral reference points are reduced. In the system investigated, this effect can be explained by the limited number and less distinctive quality of anatomical landmarks available for optical tracking. The reduced accuracy observed in edentulous jaws therefore reflects not only the scarcity of reference structures but also inherent limitations of optical tracking algorithms and dCAIS hardware. With fewer and less distinct landmarks, the registration process becomes less stable and the tracking procedure more susceptible to error. Whether a potential learning curve as previously described by other authors can compensate for this trend remains to be determined [21, 35]. At least within the present in vitro study, no clear learning effect could be observed, or it might have been covered by the degree of edentulism. Future research should aim to explore these aspects in detail.

If the present study protocol were to be applied in vivo, it remains to be seen whether deviations would increase, as previously reported by Bover-Ramos et al. in the context of CAIS [36]. It must be emphasized that specific clinical factors are not replicated in the investigated in-vitro setting. In particular, the absence of soft tissue, intraoperative bleeding, saliva, restricted visibility, and patient movements represent significant challenges in clinical reality that may negatively affect navigation accuracy [12, 22]. Therefore, while in-vitro data provide important insights, the transferability to real clinical conditions remains limited. This consideration is particularly relevant when determining appropriate safety margins during digital implant planning. Although Wei et al. recently demonstrated that deviations observed in model-based studies were generally lower than those in clinical trials, they found no statistically significant differences between the two settings [33]. As already mentioned, this is likely to be attributed to a broader visibility, better access, no interference by blood or saliva and no patient movement [37] and might represent a limitation of in vitro studies in general. Nevertheless, while in vitro studies often provide a foundational basis for subsequent in vivo research, their results should not be uncritically applied to clinical settings and must be interpreted with care.

In conclusion, methodological limitations and potential sources of bias must be acknowledged. Implant insertion was performed machine-driven using a surgical hand piece and registering the implant body with the respective registration tool. Usually, in a clinical setting, contamination of implant surfaces, which are complexly packaged under sterile conditions prior to insertion into the implant osteotomy, is minimized as much as possible. However, this contamination cannot be entirely avoided during the necessary registration process for fully guided implant placement. Therefore, it must be carefully considered whether the benefits of registration for insertion outweigh the risk of contamination. Future studies should incorporate multiple clinicians, dCAIS systems and settings with independent evaluators to enhance the objectivity and comparability of results.

Finally, the application of dCAIS usually requires clinicians to focus on a screen rather than the surgical site, which may hinder intraoperative ergonomics and hand-eye coordination. Integrating augmented/mixed reality-based dynamic navigation (AR/MR) technology as recently described by Shusterman et al. (2024) could enhance this workflow in general [38, 39]. Such an implementation could significantly enhance accuracy and usability, representing a promising direction for further digital evolution of implant surgery [1, 40].

## Conclusions

This in vitro study demonstrated that a commercially available dCAIS system can achieve comparable accuracy independent of the clinician’s experience level. The measured deviations align with previously published data. Accuracy declined with decreasing dentition. Future research should involve multiple operators and dCAIS systems including various edentulism scenarios, while exploring the integration of augmented or mixed reality technologies to enhance workflow efficiency and potentially improve accuracy. Overall, the investigated dCAIS system seems to effectively compensate for varying operators’ experience and justifies additional (pre-) clinical investigations.

## Data Availability

The datasets used and/or analysed during the current study are available from the corresponding author on reasonable request.
